# Maternal behaviours disrupted by Gprasp2 deletion modulate neurodevelopmental trajectory in progeny

**DOI:** 10.1038/s41598-024-62088-x

**Published:** 2024-05-31

**Authors:** Marta I. Pereira, Mariana Laranjo, Marcos Gomes, Mohamed Edfawy, João Peça

**Affiliations:** 1 CNC-Center for Neuroscience and Cell Biology, University of Coimbra, 3004-504 Coimbra, Portugal; 2 PhDOC PhD Program, CIBB, Faculty of Medicine, University of Coimbra, Coimbra, Portugal; 3 IIIUC-Institute for Interdisciplinary Research, University of Coimbra, 3030-789 Coimbra, Portugal; 4 PhD Program in Experimental Biology and Biomedicine (PDBEB), University of Coimbra, Coimbra, Portugal; 5 Present Address: HEMEX AG, Liestal, Switzerland; 6 Department of Life Sciences, University of Coimbra, 3000-456, Coimbra, Portugal

**Keywords:** GPRASP2, Autism, Maternal care, Ultrasonic vocalisations, Oxytocin receptor, Sex differences, Cross fostering, Sexual behaviour, Social behaviour, Neuroscience

## Abstract

Autism spectrum disorders (ASDs) are known to present sex-specific differences. At the same time, understanding how maternal behaviours are affected by pathogenic mutations is crucial to translate research efforts since rearing may recursively modulate neurodevelopment phenotype of the progeny. In this work, we focused on the effects of *Gprasp2* deletion in females and its impact in progeny care and development. Female mice, wild-type (WT), *Gprasp2*^+/−^ (HET) or *Gprasp2*^−/−^ (KO) mutants and their progeny were used and behavioural paradigms targeting anxiety, memory, maternal care, and other social behaviours were performed. Analysis of communication was carried out through daily recordings of ultrasonic vocalizations in isolated pups and cross-fostering experiments were performed to understand the effect of maternal genotype in pup development. We found that *Gprasp2*^−/−^ females presented striking impairments in social and working memory. Females also showed disruptions in maternal care, as well as physiological and molecular alterations in the reproductive system and hypothalamus, such as the structure of the mammary gland and the expression levels of oxytocin receptor (OxtR) in nulliparous versus primiparous females. We observed alterations in pup communication, particularly a reduced number of calls in *Gprasp2* KO pups, which resulted from an interaction effect of the dam and pup genotype. Cross-fostering mutant pups with wild-type dams rescued some of the early defects shown in vocalizations, however, this effect was not bidirectional, as rearing WT pups with *Gprasp2*^−/−^ dams was not sufficient to induce significant phenotypical alterations. Our results suggest *Gprasp2* mutations perturb social and working memory in a sex-independent manner, but impact female-specific behaviours towards progeny care, female physiology, and gene expression. These changes in mutant dams contribute to a disruption in early stages of progeny development. More generally, our results highlight the need to better understand GxE interactions in the context of ASDs, when female behaviour may present a contributing factor in postnatal neurodevelopmental trajectory.

## Introduction

Autism spectrum disorder (ASD) encompass a set of conditions characterised by impairments in social interactions, communication and the presence of repetitive patterns of behaviour and restricted interests^1^. With a prevalence of approximately 1 case per 100 children, ASD diagnosis shows an overall male to female ratio of 4:1^2^. Sexual dimorphisms are seen in memory, cognitive flexibility, verbal fluency, and social communication, with studies in children and adolescents showing that females are more sociable and display better fine motor skills; while exhibiting greater difficulty internalising and expressing emotions and reduced verbal control^3,4^. On the other hand, male subjects display more significant repetitive and stereotyped behaviours and greater difficulties in social skills^3,4^.

According to the DSM-5 and other literature, higher severity of symptoms and higher number of comorbidities, such as epilepsy and intellectual disability (ID), are present in females diagnosed with ASD^1,5,6^. In the cases where both ASD and ID are identified, a more proportional gender ratio is observed, hinting towards a lower likelihood of ASD diagnosis for females with normal or borderline intellectual disability^7^. The sex differences observed in both the severity of the symptomatology and bias in diagnosis have been explored in the ‘imprinted x-liability threshold model’^8^, the ‘extreme male brain theory’^9^ and the ‘female protective effect theory’^10^ as some of the rationale proposed to explain genetic and neurobiological sex differences.

Sex-effects are seen in *EphB2*^+/-^ mice where females, but not males, display memory deficits and increased repetitive behaviours^11^. *Tsc2*^+/−^ females present alterations in social interactions and more simplified communication while performing social tasks^12^ and 16p11.2^del/+^ females mice display a sex-specific increase in susceptibility to anxiety-like phenotypes following stress^13^. Recently, a study on Nf1^+/−^ mice showed a sexually dimorphic impact on hippocampal neurochemistry, but superior performance in social behaviours in females^14^. The *Mthfr*^+/−^ mice display recognition memory impairments and anxiety-like behaviours in both males and females, although with different magnitudes^15^. In contrast, the *Fmr1* knockout model has shown a lack of sex-specific effects, since both males and females present audiogenic seizures, hyperactivity, and deficits in memory^16^.

Maternal behaviours are sex-specific behaviours shown by dams during the perinatal period, which include aggression towards intruder males, pup licking and nursing, or pup retrieval to the nest. These behaviours can help reveal underlying dysfunction in innate social phenotypes. In this domain, for example, *Shank2* knockout female mice have been reported to abandon newborn pups upon delivery^17^; surrogate *Mecp2* heterozygous females show deficits in pup gathering behaviour^18^; and pups raised by *Tsc2* mutant dams display alterations in vocalization^19^.

In this study, we used a model generated via the deletion of *Gprasp2*, a gene previously linked to ASD and ID^20–23^. The G-protein Coupled Receptor Associated Sorting Protein (GPRASP) family of proteins are known to regulate the trafficking of diverse GPCRs, such as, dopamine, muscarinic and glutamatergic metabotropic receptors^24^. We previously showed that GPRASP2 modulates synaptic maturation, hippocampal function, and behaviour. We found that deletion of *Gprasp2* in male mice produced deficits in social behaviours, memory impairments and led to weight increases^25^.

Here, we show that *Gprasp2* females replicate the deficits previously observed in *Gprasp2* KO male mice in terms of memory, social interactions and weight alterations and additionally, display changes in social attachment and maternal care. We find that deletion of *Gprasp2* leads to a significant impairment in maternal care during the post parturition phase, which impacts development at early stages.

## Methods

### Mice

*Gprasp2* mutant mice generation and genotyping methods were previously described in detail in Edfawy et al.^25^. Mouse cages were maintained at a constant temperature (22 °C) and humidity (60%), under a 12 h light/dark cycle (lights on from 7 am to 7 pm). Animals were allowed access to water and food ad libitum. Female animals, ages between 6 and 14 weeks, were used in the experiments performed in this study unless otherwise noted. Maintenance and handling of the animals was performed in compliance with all relevant ethical regulations for animal testing and research, including the guidelines of the Animals Use and Care Guidelines issued by FELASA and European Directives on Animal Welfare. All experiments were carried out under animal testing research protocols approved by ORBEA (Institutional Animal Welfare Body of the University of Coimbra/CNC, reference number 282/2021) and DGAV (Portuguese Regulatory Agency, reference number 8212/2021). Heterozygous (*Gprasp2*^+/-^) dams were bred with wild-type males (*Gprasp2*^+/y^) to produce hemizygous knockout males (*Gprasp2*^-/y^), wild-type males (*Gprasp2*^+/y^) and females (*Gprasp2*^+/+^) and heterozygous females (*Gprasp2*^+/−^). Heterozygous (*Gprasp2*^+/−^) dams were bred with knockout (*Gprasp2*^-/y^) males to produce hemizygous knockout (*Gprasp2*^-/y^) and wild-type males (*Gprasp2*^+/y^) along with heterozygous (*Gprasp2*^+/-^) and homozygous knockout females (*Gprasp2*^-/-^). All behavioural tests and quantifications were performed by trained experimentalists blinded to the animal genotype. The estrous cycle was determined for a subset of animals and no significant correlation was found between behaviour and cycle stage.

This study is reported in accordance with ARRIVE guidelines.

### Open field

The test was performed as previously described in Edfawy et al.^25^. Open field consisted of an opaque arena (40 × 40 × 30 cm) and mice were automatically video tracked using Ethovision XT (Noldus, Netherlands). Mice were placed at the corner of the apparatus and locomotor behaviour was recorded for 1 h. Indirect and homogeneous illumination of the room was provided by white LED lamps at 100 lx on the centre of the maze. Time spent in the centre zone (15 cm × 15 cm) and total distance travelled were evaluated using Ethovision XT (Noldus, Netherlands).

### Elevated plus maze

The elevated plus maze consisted of an arena composed of four arms (30 × 5 cm), two of them open, the other two closed (walls 15 cm high). The animals were placed in the arena and left to freely roam for 10 min. On the two open arms, besides playing with the natural aversion mice show for open and elevated places, brightness is also added as an anxiogenic factor, with illumination of 100 lx. Mice were tracked using Ethovision XT (Noldus, Netherlands) and time spent on the open arms was evaluated.

### T-maze test

The test was performed as previously described in Edfawy et al.^25^. Briefly, the apparatus consisted of a T-shaped maze (72 × 10 cm, TS0701-M, OpenScience) elevated from the floor (60 cm). Fresh bedding was added to the maze before testing the animals. The mice were placed in the initial part of the stem and allowed to explore the maze. After the animal entered one of the arms, a sliding door was placed in the initial part of the arm chosen, allowing the mouse to explore the chosen arm. After a 30 s retention period, the animal was gently removed and returned to the homecage. After another 30 s period, the animal was returned to the start arm and a second run was initiated. Directions of choice were recorded for each mouse and the percentage of alternation obtained. Five trials (two runs each) were conducted in the space of two consecutive days (three on the first day and two on the second). The floor in the maze was homogeneously illuminated at 15–20 lx.

### Three-chamber social interaction test

The three-chamber arena was from Stoelting (Stoelting, Ireland). Mice were tested for voluntary social interaction as previously described^26^. The assay consisted of three sessions: the first session began with a 20-min habituation period during which the subject mouse freely explored all three chambers; next, the mouse was confined to the centre chamber and an empty wire cage (Empty) and a cage with an unfamiliar mouse (WT stimulus, Social Partner) were introduced to the side-chambers; in the second session, the subject mouse was then allowed to freely explore all three chambers for 20 min. Before the third and last session, the subject mouse was gently guided to the centre chamber while the empty wire cage was replaced with a caged WT stimulus mouse (Novel Partner). In the last session, the subject mouse was then left to explore all three chambers for 10 min. Stimulus mice were age-matched wild-type females previously habituated to the wire cages. The positions of the empty cage and “Social Partner” were alternated between tests. No position bias was observed. Time spent in close proximity was calculated using the automated software Ethovison XT (Noldus, Netherland). Preference index was calculated as the time spent in close proximity with the Novel/Social Partner divided by the sum of the time spent in close proximity to the Novel and Familiar/Empty partner.

### Dyadic social interaction test

Mice were tested for reciprocal social interaction, as previously described^26^. Age-, sex- and weight-matched C57BL/6 females unfamiliar with the tested mice were used as stimulus partners. The test was performed in an open arena (40 × 40 × 30 cm) filled with fresh bedding. Illumination on the arena floor was kept at 30 lx during the test and the chamber was cleaned with 70% ethanol between trials. The target mouse was removed from the homecage and placed on one side of the chamber and separated by a solid partition from the matched partner. After the 10-min acclimatisation period, the barrier was removed, and social interactions were recorded for 30 min. Social interactions (sniffing, allogrooming, chasing) were quantified, being a second run-through performed where the interactions initiated by the tested animal were identified and differentiated. These behaviours were scored manually by observers blinded to the genotype of the animals using the Observer XT 9 software (Noldus, Netherland).

### Marble burying test

The animals were placed in a new standard homecage (20 × 26 × 13 cm) filled with corn bedding up to 5 cm in height. 24 marbles were orderly and equally distanced on top of the bedding. The animals were placed in the cage and left to freely roam for 30 min. The number of marbles that were more than 25% visible were counted as unburied by at least three different observers blinded to the experimental conditions.

### Nest building test

Mice were individually housed in an unfamiliar homecage (20 × 26 × 13 cm) with corn bedding and without environmental enrichment. In each cage, a single nestlet was added at 5 pm. Nest quality produced after 16 h was recorded for each mouse and assessed by at least three observers blinded to the experimental conditions following a 5-point rating scale as described in^27^.

### Pup retrieval test

The retrieval assay was performed in the homecage of the dam. From PND2 to PND4, identical sessions were performed daily. On each test day, the pups were separated from the dam for 30 min prior to the test and kept warm during the separation, maintaining the nest untouched inside the cage. Immediately prior to the assay the dams were briefly removed from the homecage and three of the pups were placed in each corner of the cage except for the one closest to the nest. The dam was then reintroduced to the cage, being placed in the nest facing the wall. The latency for the female to retrieve all three pups to the nest was assessed. If after 10 min there would be pups still out of the nest, the test would be stopped, the remaining pups were retrieved and placed in the nest. In these instances, the latency score attributed was 600 s. All females assessed were primiparous females, having no previous gestation and maternal care experience.

### Maternal aggression test

The test was performed on postnatal day 5 in the homecage of the dam. The pups were removed from their cage, 3 min before the start of the test to prevent infanticide, but the nest was left in place. For the test, sexually naïve C57BL/6 males were introduced to the cage and the interactions were recorded for 10 min. The latency to first attack, the number of aggressive bouts and the duration of these behaviours were assessed. Chasing, biting, and wrestling were considered aggressive behaviours/ attacks. These behaviours were scored manually by observers blinded to the genotype of the animals using the Observer XT 9 software (Noldus, Netherland). All females assessed were primiparous females, having no previous gestation and maternal care experience.

### Ultrasonic vocalisations recordings

Isolation-induced ultrasonic vocalisations (USVs) were recorded from PND2 to PND15. USVs were recorded for 6 min in a sound-attenuated styrofoam box. The microphone was placed through a hole in the middle of the cover of the styrofoam box, about 6 cm above the pup which was placed in a small petri dish. Recordings were obtained using the UltraVox XT system (Noldus Information Technology, The Netherlands), which could record the full spectrum of sound with a maximum frequency of 160 kHz. After recording USVs, pups were returned to the homecage. Between PND0 and PND1, the pups were marked on the paws to ensure that each pup was only recorded once daily.

All the USV recordings were analysed offline with the Ultravox software using a 250 kHz sampling rate and a maximum frequency of 120 kHz. The USVs were analysed manually by a researcher blind to the phenotype. The USVs were then distinguished manually within 10 different subtypes based on their qualitative and quantitative characteristics, according to the categories established by^28^. “Short” calls had a length of less than 5 ms; “Flat” calls had inflections of less than 3 kHz; “Downward” calls showed a continuous decrease in pitch; “Upward” calls showed a continuous increase in pitch; “Chevron” calls presented an inverted U-shape with an increase in frequency followed by a decrease; the “Complex” class exhibited one call containing two or more directional changes in frequency; Two-syllable calls displayed two components: a main call (flat or downward) and an additional short call with higher frequency in the end; “Composite” calls were composed of two calls, at different frequencies, emitted simultaneously; Frequency steps presented instantaneous frequency steps showing a vertical discontinuous step on a spectrogram with no interruption in time; Harmonics presented one main call but with additional calls of different frequencies surrounding it.

### Cross fostering experiments

To carry out cross fostering experiments, programmed mating sessions were performed and litters born within 12 h of each other, originated from different genotyped females, were used. At PND0, the size and gender prevalence within the litters was assessed. Before switching the pups between Gprasp2^+/+^ and Gprasp2^−/−^ dams, litters were culled to set the number of pups within each litter and the gender of pups between the two litters being tested. To minimise rejection from the dams, pups were maintained in heating pads during this process and placed in bedding from the foster dam prior to being placed in a novel cage. At PND2, PND8 and PND15 the pups were subjected to the ultrasonic vocalisation recording protocol, as described above. The pups were also handled and weighed daily.

### Tissue collection

Animals were anaesthetised using isoflurane (IsoVet) and euthanized by decapitation. The different tissues (specified in Table 1) were collected and dissected on ice and immediately stored at – 80 °C, until further processing.

**Table 1:** Characteristics of the different tissues collected along with their purpose.

Gender/age	Tissue collected	Technique	Target
Female/2 months	Whole brain	Western blot	Gprasp2 (1:1000, Proteintech) β-actin (1:10,000, Sigma-Aldrich)
Female/1 month	Hypothalamus, Cortex, Cerebellum, Striatum, Hippocampus	Western blot	Gprasp2 (1:1000, Proteintech) β-actin (1:10,000, Sigma-Aldrich)

### Biochemical analysis

For protein extraction, RIPA Lysis buffer (150 mM NaCl, 1% Triton X-100, 0.5% DOC, 0.1% SDS, 50 mM Tris pH 7.4) was added to the samples. Manual homogenization was performed until no clear tissue was visible followed by a sonication protocol (15 secs on, 30 secs off, 3 times) always keeping the samples refrigerated. Samples were then centrifuged at max rotation (g) for 20 min at 4 °C (Heraeus Fresco 21 Centrifuge, ThermoFisher). Protein quantification was performed using the BCA protein assay kit (Pierce BCA protein assay Kit, Thermo Scientific), following the manufacturer’s instructions. Protein samples (55 μg) were denatured with 4 × Laemmli sample buffer (Bio-Rad) and 10% β-mercaptoethanol. Samples were incubated at 95 °C for 5 min before western blotting.

### Western blotting

Equal amounts of proteins were resolved by SDS-PAGE in 10% polyacrylamide gels. Proteins were transferred to PVDF membranes Immobilon-P (Millipore), blocked at room temperature (RT) for 1 h in 1 × TBS + 0.1% Tween-20 (TBS-T) with 5% milk. Membranes were incubated with primary antibodies (diluted in TBS-T with 0,5% milk) at 4 °C overnight before washing in TBS-T, 3 times for 15 min each, and incubated with secondary antibodies (1:10,000 anti-mouse, anti-rabbit, Jackson ImmunoResearch) for 120 min at room temperature. Following three-time washes in TBS-T, the membranes were incubated with ECF western blot substrate (Vistra ECF, Sigma-Aldrich).

Signal was visualised on ChemiDoc Imaging System (Biorad). Antibodies used in western blotting experiments were the following: anti-GPRASP2 (12,159–1-AP, 1:1000; Proteintech). For all experiments, anti-β-actin (A5441, 1:10,000; Sigma-Aldrich) antibody was used as loading control.

### qRT-PCR

Total RNA was extracted from hypothalamus collected from both nulliparous (4 months old) and primiparous females (4 months old, 15 days post-delivery, before weaning) with the use of the NucleoSpin RNA kit and according to the instructions of the supplier (Macherey–Nagel). RNA extracted (1.15 μg) was retrotranscribed to complementary DNA using the NZY First-Strand cDNA Synthesis kit (NZYTech), following the manufacturer’s instructions. The synthesised complementary DNA was diluted 15 × in RNAse-free water and stored at − 20 °C. To avoid genomic DNA contamination a DNase I treatment step was performed during the RNA extraction and the primers’ design (specified in Table 2) targeted regions between adjacent exons, when possible. Primer specificity was verified by the melting curve shape, peak temperature, and the amplification of a single product with the expected size confirmed by electrophoresis in an agarose gel.

**Table 2:** Primer sequences used for qRT-PCR reactions**.**

Target gene	Forward primer (5’- > 3’)	Reverse primer (5’- > 3’)
Gprasp2	TGATTTCAGTCTTGAGCCGC	GATGAGTGGCAGGTGGTTAA
Oxt	GGAGAACTACCTGCCTTCG	GAAGCGCGCTAAAGGTATTC
OxtR	GGAGCGTCTGGGACGTCAAT	AGGAAGCGCTGCACGAGTT
Hprt	GCTTACCTCACTGCTTTCCG	CATCATCGCTAATCACGACGC

In the case of the oxytocin receptor, an extra step was added given the low concentration values of this product. For this, a pre-amplification step was added. This step consisted of the following PCR protocol: denaturation step for 2 min at 95 °C followed by 14 cycles of amplification (10 s at 95 °C and 4 min at 60 °C). For this, a 50 μL reaction mix containing 5 units of MyTaq DNA Polymerase (Bioline), 0.015 mM of specific primers (forward and reverse primers for both *OxtR* and *Hprt*), 10 μL of 5 × MyTaq Reaction buffer (Bioline), 2 μL of the previously transcribed cDNA in RNAse free water. The resulting product was then used in the following PCR reaction.

For the final PCR reaction, a mix containing 5 μL of NZYSpeedy qPCR Green Master Mix (NZYTech), 1 μL of forward and reverse primer mix (10 μM each) and 4 μL of complementary DNA was added to each well. The reaction was initiated with a polymerase activation step for 30 s at 95 °C. After, 40 cycles of a 5 s denaturation step at 95 °C followed by 20 s of an annealing/extension step at the optimal primer annealing temperature of 60 °C were performed. After 40 cycles of amplification, a melting curve protocol was performed. All reactions were performed in duplicates. Quantitative PCR assays were performed and analysed with a real-time PCR system (StepOnePlus, Applied Biosystems, ThermoFisher Scientific, USA). The comparative CT method (DDCT) was used to quantify the relative gene expression of each sample, using *Hprt* as the endogenous reference gene. The endogenous reference sample used corresponded to the *Gprasp2*^+/+^ nulliparous females.

### Mammary gland whole-mount staining

Abdominal mammary glands were collected and placed on a gelatinated microscope slide. The glands were manipulated and spread using blunt tweezers and immediately placed in Kahle’s fix solution overnight. The glands were then washed in 70% ethanol for 15 min, gradually changed and rinsed with distilled water for 5 min. They were then stained overnight in a carmine alum staining solution. The glands were afterwards washed for 15 min in 70% ethanol, 95% ethanol and 100% ethanol in order. The glands were then cleared in xylene for 72 h (or until the fat pads appeared transparent) at which point they were mounted using Richard-Allan scientific mounting medium (Thermo Scientific). Slides were stored at room temperature until further analysis. Whole mammary gland images were acquired in an Axio Imager Z2 microscope (Zeiss, Germany) with an EC Plan Neofluor 10x/0,3 lens. Each image consisted in a compilation of tiles encompassing the whole tissue. A crop of 1500 × 1500 pixels in size was utilised as a representation of the whole tissue. Tracing reconstruction was performed using the NeuronJ plugin in ImageJ, extracting the total length of the vasculature in each crop. Both abdominal mammary glands were analysed, and a mean of both sides was used when plotting the data. Experiments were performed blind to the animal’s genotype both during image acquisition and analysis.

### Statistical analysis

Data is represented as mean values ± SEM. Statistical analysis was performed using parametric (one sample t-test) and non-parametric tests (two-tailed Mann–Whitney test, two-way repeated measures ANOVA), followed by post hoc analysis when appropriate, as indicated in figure legends. Analysis was performed using Graphpad (Prism) or MATLAB (Mathworks). Subject randomisation was performed. Sample size calculation were performed using G*Power and considering the authors prior experience. Statistical significance was defined as ****p < 0.0001, ***p < 0.001, **p < 0.01, *,#*p* < 0.05.

### Ethics approval

All relevant ethical approvals were collected and include ORBEA (Institutional Animal Welfare Body of the University of Coimbra/CNC, reference number 282/2021) and DGAV (Portuguese Regulatory Agency, reference number 8212/2021).

## Results

**Figure 1:**
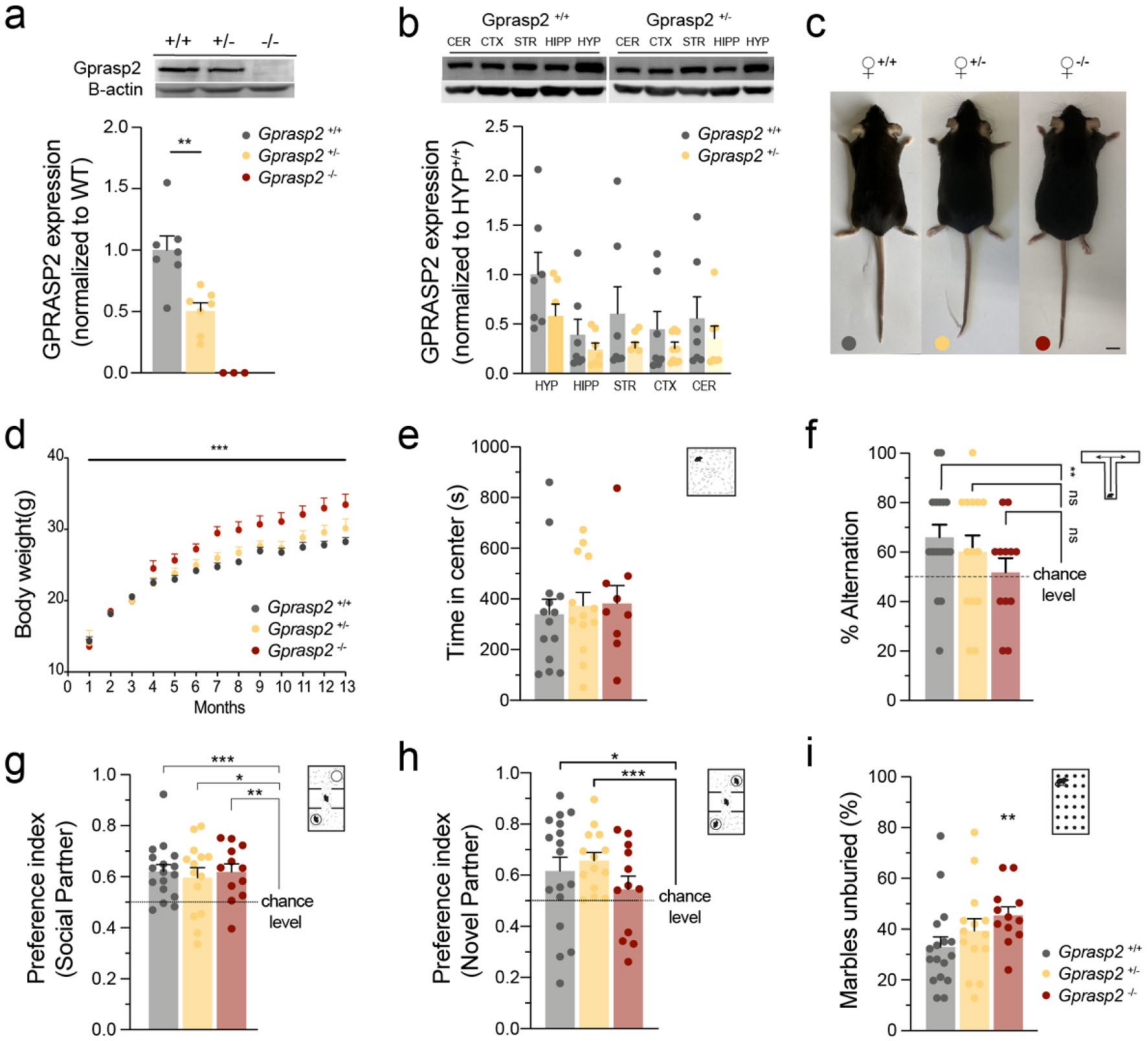
*Gpraps2* mutant female mice display increased body weight and deficits in memory and social behaviours. (**a**) Western blotting of 2-month-old whole brain samples shows decreased expression of GPRASP2 in heterozygous females and protein deletion in *Gprasp2*^-/-^ when compared with wild-type littermates, WT n = 7, HET n = 7, KO n = 3, two-tailed Mann–Whitney test. (**b**) GPRASP2 is highly enriched in the hypothalamus of 1 month-old *Gprasp2*^+/+^ and *Gprasp2*^+/-^ females, n = 7, two-tailed Mann–Whitney test. (**c**) Representative images from 1 year old WT, HET, and KO female mice (scale bar represents 1 cm). (**d**) *Gprasp2*^-/-^ females present an increase in body weight starting approximately at 4-months of age becoming more striking throughout adulthood; WT n = 18, HET n = 23, KO n = 13, two-way repeated measures ANOVA. (**e**–**i**) *Gprasp2* deficient females present memory and social impairments. (**e**) *Gprasp2* deficient females show no significant alterations in the open field test; WT n = 14, HET n = 13, KO n = 9, two-tailed Mann–Whitney test. (**f**) In the t-maze for spontaneous alternation, *Gprasp2*^-/-^ and *Gprasp2*^+/-^ females display impaired alternation when compared with control mice; WT n = 17, HET n = 14, KO n = 12; One sample t test against hypothetical value of chance alternation at 50%. (**g**) *Gprasp2*^-/-^ and *Gprasp2*^+/-^ females show no impairments in the sociability component of the three-chamber social test; WT n = 17, HET n = 14, KO n = 12; One sample t test against hypothetical value of chance preference at 0.5. (**h**) In the social novelty component of the three-chamber test, *Gprasp2*^-/-^ females display reduced preference index for a novel social partner when comparing to WT controls; WT n = 17, HET n = 14, KO n = 12; One sample t test against hypothetical value of chance preference at 0.5. (**i**) *Gprasp2*^+/-^ and *Gprasp2*^-/-^ females show a slight and significant, respectively, increase in the percentage of marbles unburied in the marble burying test; WT n = 17, HET n = 14, KO n = 12, two-tailed Mann–Whitney test. All data are presented as means ± s.e.m. Statistical significance: *p < 0.05 and **p < 0.01, ***p < 0.001, ****p < 0.0001 (CER-cerebellum; CTX-cortex; STR-striatum; HIPP-hippocampus; HYP-hypothalamus).

We started by performing a western blot analysis to confirm gene-dose dependence of protein expression in females of all genotypes (Fig. 1a). As expected, heterozygous females for *Gprasp2* show an overall decrease of approximately 50% in the expression of the protein in 2-month-old whole brain tissue samples (Fig. 1a). When assessing the expression of GPRASP2 in the brain, we observe this protein is highly enriched in the hypothalamus in both wild-type and heterozygous females (Fig. 1b). In terms of overall morphology, *Gprasp2*^−/−^ (KO) and *Gprasp2*^+/−^ (HET) females did not display gross abnormalities except for an increase in body weight in KO females, significant after 4-months of age, and increasingly more striking with age (F = 10.94, *p* = 0.0001, Fig. 1c and d). In this work, we studied female *Gprasp2*^+/+^, *Gprasp2*^+/−^ and *Gprasp2*^−/−^ during the juvenile period (6–14 weeks of age), before significant differences in body weight were observed that could interfere with other analyses.

Given the previously described mutations in *Gprasp2* associated with ASD and other neurodevelopmental disorders^20–23^ and the behavioural alterations previously described in *Gprasp2* KO males^25^, we assessed if females also presented behavioural alterations in a battery of tests. Initially, an assessment of anxiety-like levels was performed through an open field test (WT: 339.9 ± 58.83 s; HET: 372.5 ± 53.2 s; KO: 382.1 ± 70.75 s; Fig. 1e) and elevated plus maze (WT: 46.4 ± 6.14 s; HET: 25.2 ± 6.27 s; KO: 39.1 ± 8.84 s; Supplementary Fig. 1a). In both behavioural paradigms, no significant alterations were observed between KO or HET females with WT controls (*p* > 0.05). Additionally, no gross abnormalities were found in motor function when compared total distance travelled in the open field (WT: 11,839 ± 723.9 cm; HET: 11,195 ± 739.7 cm; KO: 11,486 ± 1298 cm; P > 0.05, Supplementary Fig. 1b). Analysis of possible alterations in working memory using the T-maze test for spontaneous alternation, showed that both heterozygous and knockout females present significant deficits in memory (WT: 65.88 ± 5.08%, t_(16)_ = 3.128, *p* = 0.0065; HET: 60.00 ± 6.63%, t_(13)_ = 1.508, *p* = 0.1554; KO: 51.67 ± 5.75, t_(11)_ = 0.2898, *p* = 0.7774; Fig. 1f). To assess for alterations in social behaviours and interactions, the three-chamber social test and the social dyadic paradigm were performed. In the three-chamber test, no major differences were observed in the social preference part of the test, as all genotypes significantly showed a preference index above 50% (WT: *p* < 0.001; HET: *p* < 0.05; KO: *p* < 0.01; Fig. 1g). However, deficits were observed in the second part of the test which assesses the response of the animals to social novelty. Here, *Gprasp2* KO females showed no preference between a novel partner and a familiar one (WT: *p* < 0.05; HET: *p* < 0.001; KO: *p* > 0.05; Fig. 1h). In the social dyadic test, HET and KO females show a trend towards increased interactions with a stimulus, but no significant differences were found when compared to WT control females (*p*_WT × HET_ > 0.05; *p*_WT × KO_ > 0.05; Supplementary Fig. 1c). In the marble burying test, an assay that often appears altered in various animal models of ASD, Gprasp*2*^-/-^ females presented an increase in the percentage of marbles unburied when compared to WT controls (WT: 32.59 ± 4.001; HET: 38.99 ± 4.763, *p*_WT × HET_ = 0.2031; KO: 45.14 ± 3.305, *p*_WT × KO_ = 0.0061; Fig. 1i). Together, this behavioural characterization suggests that *Gprasp2* KO females display a significant impairment in assays requiring memory and discrimination of social novelty. A comparison between the phenotypes observed in *Gprasp2*-KO females and our previous report on *Gprasp2* KO males^25^ is reported thoroughly in Supplementary Table 1. Most salient changes include an effect of Gprasp2 deletion on body weight increase and working memory and social memory impairments, regardless of the sex of the animals.

**Figure 2:**
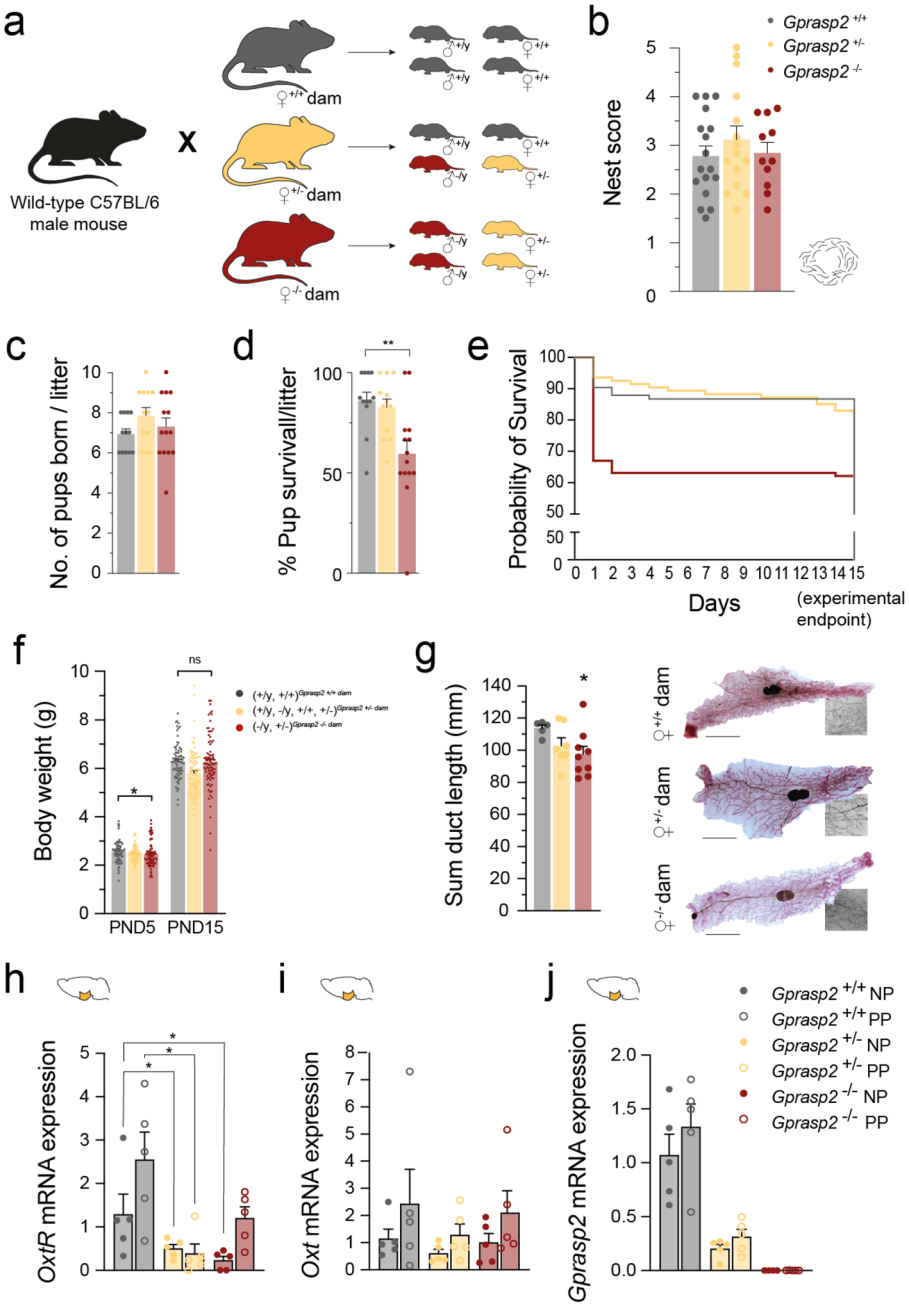
Decreased litter survival and deficits in reproductive system in *Gprasp2* mutant dams**. **(**a**) Schematic diagram of the matings established and possible genotypes of each litter depending on the female progenitor. (**b**) Females show no significant alterations in the nest building activity; WT n = 17, HET n = 14, KO n = 12, two-tailed Mann–Whitney test. (**c**) At PND0, no significant differences are seen in terms of the size of the litters from the *Gprasp2* deficient females when compared to WT controls, WT n = 12, HET n = 9, KO n = 8, two-tailed Mann–Whitney test. (**d**) Pups born from *Gprasp2*^-/-^ females have a lower chance of survival when compared to pups born from WT controls, WT n = 12, HET n = 9, KO n = 8, two-tailed Mann–Whitney test. (**e**) Majority of mortality of pups from *Gprasp2*^-/-^ females occurs during the two first postnatal days, WT n = 12, HET n = 9, KO n = 8. (**f**) Weights in the pups nurtured by dams of different genotypes throughout early development, show a small reduction at P5 in animals from KO dams, that is recovered by P15; (+ /y) ^*Gprasp2* +/+dams^ n = 38; (+ / +) ^*Gprasp2* +/+dams^ n = 42; (+ /y) ^*Gprasp2* +/-dams^ n = 21; (-/y)^*Gprasp2* +/-dams^ n = 16; (+ / +) ^*Gprasp2* +/-dams^ n = 28; (+ /-) ^*Gprasp2* +/-dams^ n = 21; (-/y)^*Gprasp2* -/-dams^ n = 47; (+ /-) ^*Gprasp2* -/-dams^ n = 40, two-tailed Mann–Whitney test. (**g**) Mammary gland duct complexity is significantly lower in glands from *Gprasp2*^-/-^ females (representative images scale bar 5 mm) WT n = 5, HET n = 7, KO n = 9, two-tailed Mann–Whitney test. (**h**) Oxytocin receptors in the hypothalamus show decreased mRNA expression in both KO and HET nulliparous (NP) and primiparous (PP) females when compared to their WT controls, n = 5/group, two-tailed Mann–Whitney test. (**i**) No significant alterations are observed between genotypes in terms of mRNA levels of oxytocin in the hypothalamus of these females, being a tendency for increase present in all genotypes when comparing PP to NP females, n = 5/group, two-tailed Mann–Whitney test. (**j**) *Gprasp2* seems to be slightly increased in PP females when compared to NP females, n = 5/group, two-tailed Mann–Whitney test. All data are presented as means ± s.e.m. Statistical significance: *p < 0.05 and **p < 0.01.

Given the impact of social bond between dams and their offspring in early development, we decided to focus on the effect of *Gprasp2* deletion on maternal care and the consequence to their offspring. To achieve this, females of the three genotypes were crossed with C57BL/6 males as illustrated in Fig. 2a, and their behaviours after parturition were explored. We found no gross alterations in nest building performance and fertility (Fig. 2b and c). However, while no significant changes were observed in the number of pups present at PND0 (postnatal day 0, day of delivery) (Fig. 2c), a significantly lower number of pups from *Gprasp2*^-/-^ dams survived until PND15 (Fig. 2d). The percentage of pup survival was close to 90% for the litters of primiparous WT and HET dams. However, litters from primiparous KO dams showed a significantly lower percentage, with only 60% of the pups surviving beyond PND2 (Fig. 2e). Looking into the body weight of the pups throughout early development, a small decrease in pups from KO dams was seen at PND5, however, these changes were no longer present at PND15 (WT_PND5_: 2.566 ± 0.04872 g; KO_PND5_: 2.458 ± 0.05425 g; *p* < 0.05; WT_PND15_: 6.298 ± 0.08329 g; KO_PND15_: 6.271 ± 0.1187 g; *p* > 0.05, Fig. 2f). These data, however, contrasts with the significant weight gain seen in adult, male or female, GPRASP2 mutant mice. To further explore the drop in pup survival at such an early stage, we performed an analysis of mammary gland morphology. We found that prior to birth, *Gprasp2* KO females already presented a significant decrease in the length of the ducts in the mammary gland (WT: 113.3 ± 2.046 mm; HET: 102.5 ± 4.83 mm; KO: 97.14 ± 4.856 mm, *p*_WT x KO_ = 0.029; Fig. 2g). Given that milk production could be impaired not only by constraints in gland physiology, but also considering the role of oxytocin receptors in this process, we performed quantitative PCR analysis of hypothalamic samples collected from both nulliparous and primiparous females (before weaning) to assess possible alterations in the expression of oxytocin and its receptor. Using qPCR analysis in hypothalamic samples, we found that *Gprasp2*^-/-^ nulliparous females presented a significant decrease in the *OxtR* levels when compared to WT controls (WT: 1.288 ± 0.4673; HET: 0.4998 ± 0.0946; KO: 0.2285 ± 0.09462, *p*_WT × KO_ = 0.0317; Fig. 2h). Regarding the mRNA levels for oxytocin, we found a slight increase between primiparous females when compared to nulliparous, but no changes between genotypes (*p*_WT-NP × HET-NP_ > 0.05; *p*_WT-NP × KO-NP_ > 0.05; Fig. 2i). Besides the expected decrease in *Gprasp2* mRNA expression, we also found a trend for increased *Gprasp2* expression following parturition in both wild-type and heterozygous females (Fig. 2j).

**Figure 3:**
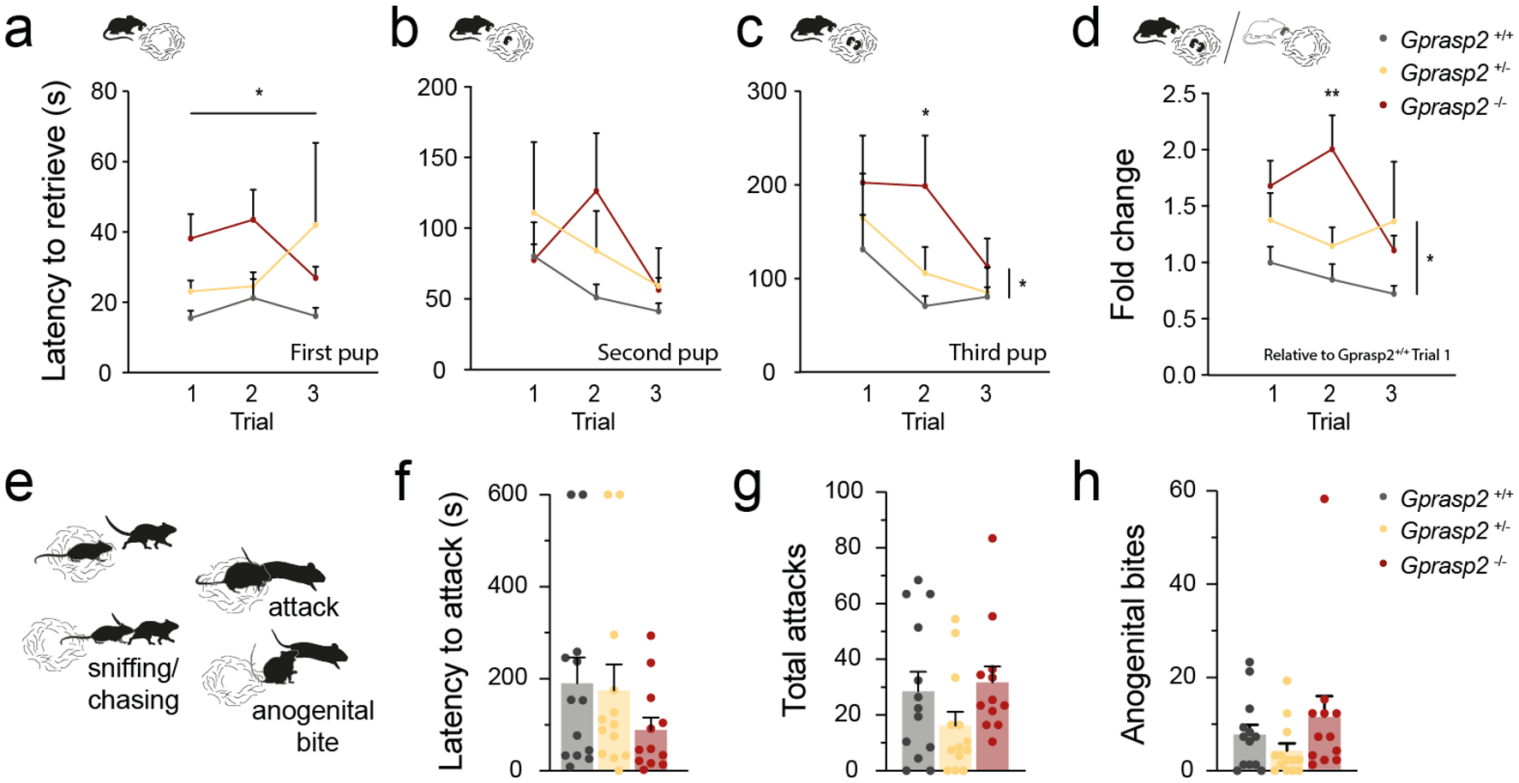
*Gprasp2* knockout impairs maternal behaviour. (**a**-**d**) *Gprasp2*^-/-^ females show a significant increase in latency in retrieving the first (**a**) and third pup (**c**), being this increased latency still seen although not as strikingly for the second pup (**b**). Overall, *Gprasp2*^-/-^ females show an increased latency to retrieve pups when compared to WT controls (**d)** data normalised to Gprasp2^+/+^ females, trial 1 relative to each pup), WT n = 12, HET n = 11, KO n = 14, two-way repeated measures ANOVA. (**e**) Schematic diagram of some of the behaviours analysed in the maternal aggression test**. **(**f–h**) *Gprasp2*^-/-^ females show a tendency for increased aggression with a slight decrease in latency to attack the male intruder (**f**) with no difference in terms of total number of attacks (**g**) and slight increase in number of anogenital bites (**h**), two-tailed Mann–Whitney test WT n = 13, HET n = 13, KO n = 12. All data are presented as means ± s.e.m. Statistical significance: *p < 0.05 and **p < 0.01.

We then studied if maternal care provided by the dams was impaired in the pup retrieval test. Here, *Gprasp2*^-/-^ dams presented a significant increase in the latency to retrieve the first pup (F = 3.796, *p* = 0.0257) and the third pup in all the three trials (F = 3.633, *p* = 0.03), but no significant changes in the retrieval of the second pup (F_(2, 102)_ = 1.151, *p* > 0.05), when compared to WT dams (Fig. 3a-c). Overall, normalising the values to latency to the first trial, we observe that both KO and HET dams presented a higher latency to retrieve pups in all three trials, and KO dams exhibited a significantly higher latency when compared to WT controls (F = 7.267, *p* = 0.0008; Fig. 3d). During the peripartum period, female mice display innate protective behaviours and aggression particularly towards male intruders^29^, which can be assessed with the maternal aggression test (Fig. 3e-h). However, we found no significant differences between dams of either genotypes in either latency to attack (Fig. 3f), total number of attacks (Fig. 3g) or number of anogenital bites (Fig. 3h). Together, these data show that Gprasp2 knockout dams display an alteration in pup-directed behaviours, but not in aggressive responses towards intruders.

**Figure 4:**
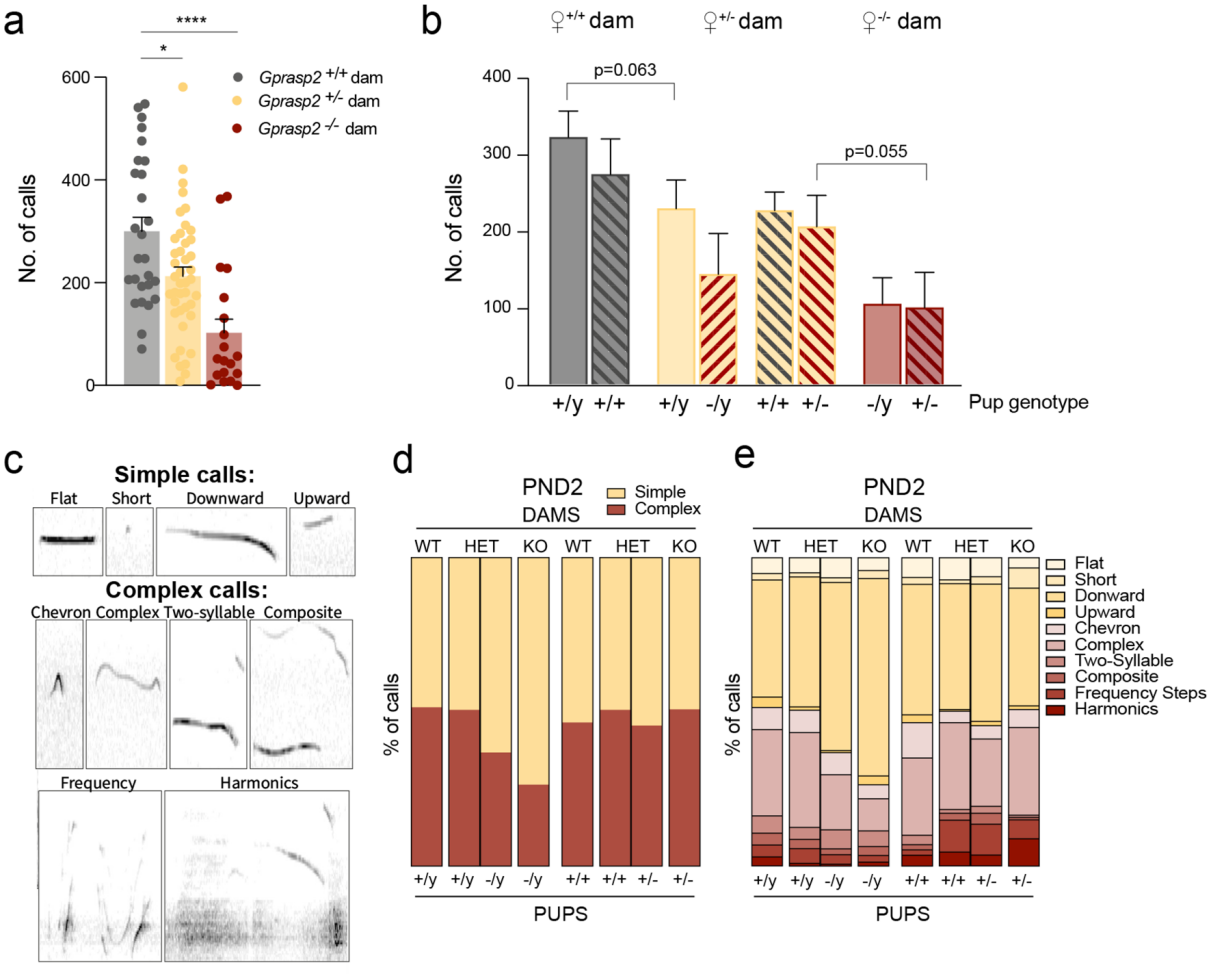
Reduced number and complexity of calls in male pups reared by knockout dams. (**a**) At PND2, pups from *Gprasp2*^-/-^ and *Gprasp2*^+/-^ dams show a significant decrease in overall vocalisations, WT n = 4 dams, HET n = 6 dams, KO n = 4 dams, two-tailed Mann–Whitney test. (**b**) The decrease is present in both males (-/y) and females (+ /-) at this stage when compared with calls from wild-type pups from *Gprasp2*^+/+^ and *Gprasp2*^+/-^ females, two-tailed Mann–Whitney test. (**c**) Examples of the different categories within which the calls were placed and their two broad groups (simple and complex). (**d**) When looking into the characteristics of these vocalisations, a decrease in terms of the complexity of the vocalisations is observed, at PND2, in pups from *Gprasp2*^-/-^ and *Gprasp2*^+/-^ dams, especially in the *Gprasp2*^-/y^ males. (**e**) Unravelling the characteristics of these calls even further, a higher predominance of downward calls can be observed at PND2 in the males from *Gprasp2*^-/-^ dams when compared to WT controls. (+ /y)^WT dams^ n = 15; (+ / +)^WT dams^ n = 12; (+ /y)^*Gprasp2* +/-dams^ n = 13; (-/y)^*Gprasp2* +/-dams^ n = 5; (+ / +)^*Gprasp2* +/-dams^ n = 14; (+ /-)^*Gprasp2* +/-dams^ n = 9; (-/y)^*Gprasp2* -/-dams^ n = 12; (+ /-)^*Gprasp2* -/-dams^ n = 7, two-tailed Mann–Whitney test. All data are presented as means ± s.e.m. Statistical significance: *p < 0.05 and ****p < 0.0001.

In the case of rodents, ultrasonic vocalisations (USVs) are important for communication between the dams and the pups in early stages of development^30–32^. USVs can trigger maternal care and facilitate communication between the mother and their offspring^31,33^. To better understand this, we analysed USV emissions from isolated pups, at three different stages of development, PND2, PND8 and PND15. At PND2, pups from KO and HET dams, regardless of their genotypes, showed a significant decrease in the number of calls emitted when compared to pups from WT-control dams (WT: 300.1 ± 27.73; HET: 212.5 ± 18.14, *p*_WT × HET_ = 0.0213; KO: 102.5 ± 26.81, *p*_WT × KO_ < 0.0001; Fig. 4a). When looking into these differences but discriminating the genotype of the pups within the litters, we observed that the number of USVs emitted by pups from *Gprasp2* KO dams, was lower when compared to wild-type pups from both WT and HET dams (Fig. 4b). Although not statistically significant, a decrease was also observed in pups from *Gprasp2* KO dams when compared to genotype-matched pups from HET dams, and a similar effect with genotype-matched pups from HET dams to WT dams (Fig. 4b). This could suggest an effect on pup vocalisation that is dependent on the genotype of the pup and the genotype of the dam.

Next, USVs were categorised according to their complexity as represented in Fig. 4c. A broad analysis of complexity of the calls showed a significant decrease in the percentage of complex calls in male *Gprasp2*^-/y^ pups from HET and KO dams when compared to male *Gprasp2*^+/y^ pups from WT dams ((+ /y)^WT^: 51.45 ± 2.408%; (−/y)^HET^: 36.79 ± 7.332%, *p*_(+/y) WT × (-/y) HET_ = 0.0328; (-/y)^KO^: 24.17 ± 6.732%, *p*_(+/y) WT x (-/y) KO_ = 0.0025; Fig. 4d). However, we found no alteration in call complexity in females from either WT, HET or KO dams (Fig. 4d). Using a subdivision of USVs into ten categories previously defined by Scattoni et al*.*^28^, we could observe an increase in the percentage of simple calls, especially downward calls, emitted by Gprasp2^-/y^ pups from both HET (54.54 ± 21.11%, *p* = 0.0655) and KO dams (63.96 ± 21.45%, *p* > 0.001) when compared to WT controls (37.97 ± 7.19%) (Fig. 4e). At PND8, no significant differences were observed in the number of calls between the offspring of WT and KO dams although a significant increase is seen in the number of calls from offspring of HET dams (Supplementary Fig. 2a). Regarding the complexity of these calls, a male-specific decrease is observed in *Gprasp2*^+/y^ pups from HET dams and *Gprasp2*^-/y^ pups from KO dams when compared to *Gprasp2*^+/y^ pups from WT dams ((+ /y)^WT^: 62.52 ± 2.958%; (+ /y)^HET^: 50.48 ± 5.605%, P_(+/y) WT x (+/y) HET_ = 0.0292; (-/y)^KO^: 49.85 ± 5.67%, P_(+/y) WT x (-/y) KO_ = 0.1028; Supplementary Fig. 2b-c). At PND15, no significant differences were observed in the number or complexity of USVs (Supplementary Fig. 2a-c).

**Figure 5:**
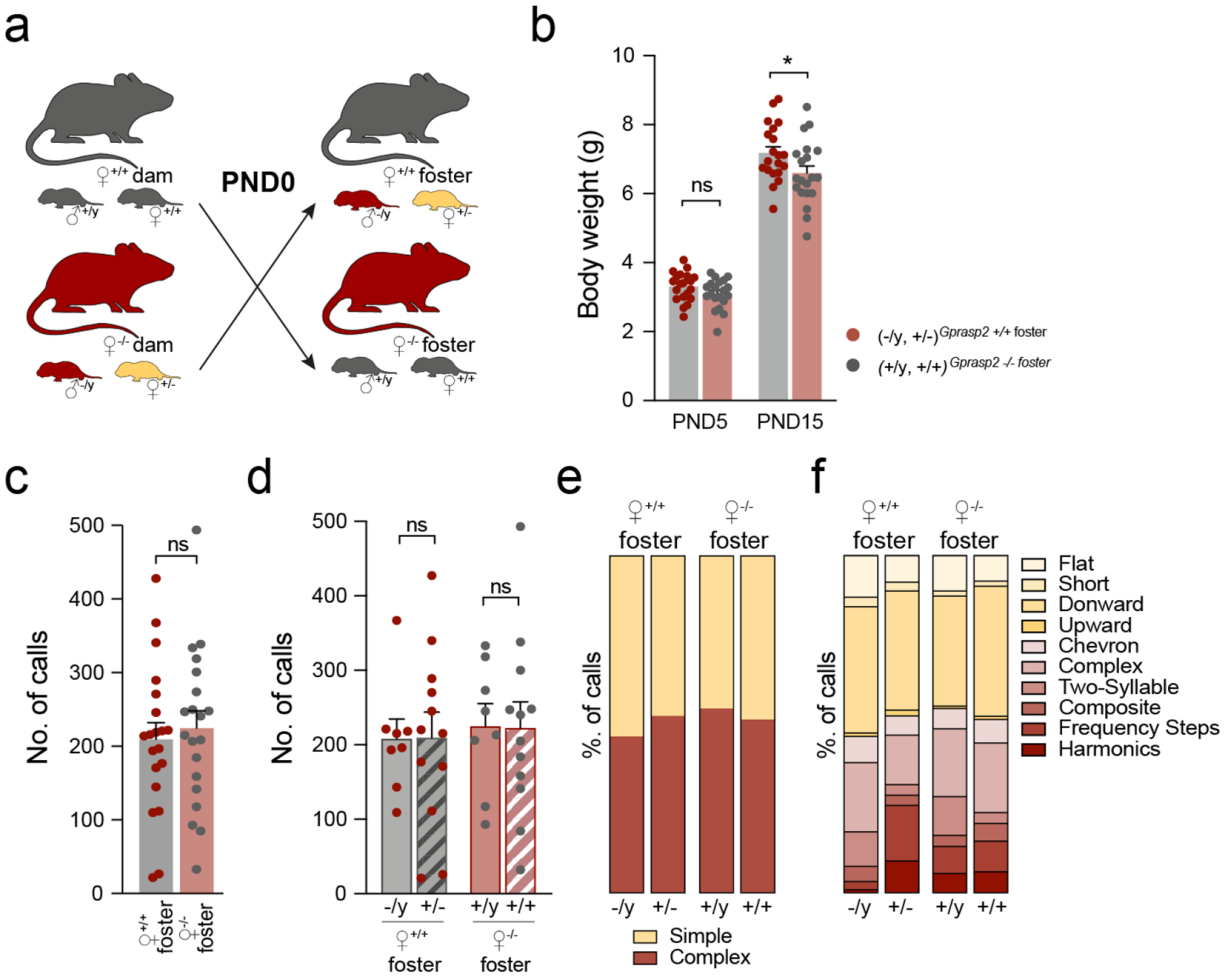
Cross-fostering by wildtype dams rescues neurodevelopmental trajectory in mutant male mice. (**a**) Schematic representation of the cross-fostering experiment. (**b**) *Gprasp2*^-/y^ pups fostered by *Gprasp2*^+/+^ dams show an increase in body weight at PND15 when compared to wild-type males fostered by *Gprasp2*^-/-^ dams, while no differences are observed at PND5, two-tailed Mann–Whitney test. (**c**) Cross-fostering led to a normalisation of the number of calls at PND2, the pups showing a similar number of calls with both fosters, two-tailed Mann–Whitney test, two-tailed Mann–Whitney test. (**d**) The cross-fostering effect in the normalisation of the number of calls is not sex-specific with both males and females from both genotypes showing alterations, two-tailed Mann–Whitney test. (**e**) In terms of call complexity, at PND2, the significant differences previously observed with the normal crossing are also lost, the pups showing similar percentages when cared for by the fosters, two-tailed Mann–Whitney test. (**f**) As for call characterization, no significant alterations are observed between pups independently of sex and foster genotype (+ /y) n = 8; (+ / +) n = 12; (-/y) n = 8; (+ /-) n = 12, two-tailed Mann–Whitney test. All data are presented as means ± s.e.m. Statistical significance: *p < 0.05.

To disentangle the effect of dam-genotype and pup-genotype interaction in the USV number and complexity, we decided to perform a cross-fostering experiment. To do so, litters from *Gprasp2* WT and *Gprasp2* KO dams were switched at PND0, as illustrated in Fig. 5a. Daily weighting of the pups revealed that litters fostered by WT dams showed an increase in body weight at PND15, when compared to WT pups fostered by KO dams (KO_fostered pups_: 6.599 ± 0.2076 g; WT_fostered pups_: 7.180 ± 0.1833; *p* < 0.05; Fig. 5b). When contrasted to the weight in the normal breedings (Fig. 2f), and the weight gain in adult GPRASP2 mutant mice, this data suggests that weight gain in mutant pups is limited when the nursing dam is performed by a KO dam.

When we analysed the effects of the cross fostering in USVs emitted by isolated pups, at PND2, we found the number of calls to be similar when assessing the data by the genotype of the foster dam (Fig. 5c). Moreover, when further analysing the genotype of the pups within the litters, no changes were observed in USV calls between WT and KO male pups when care was given by dams of opposing genotype ((+ /y)^KO^: 224.8 ± 30.65; (-/y)^WT^: 207.8 ± 26.72, *p*_(+/y) KO x (-/y) WT_ > 0.05; Fig. 5d). At this stage of development, the number of calls emitted by KO pups fostered by WT dams is increased and the opposite occurred for pups fostered by KO dams, when comparing the absolute values from fostered pups with the same pups cared for by their biological dams (((+ /y)^WT^: 321.7 ± 34.00, *p*_(+/y) WT × (+/y) KO_ = 0.1536; (-/y)^KO^: 104.3 ± 34.48, *p*_(-/y) WT × (-/y) KO_ = 0.0691; see Fig. 4a and 5c). When comparing the complexity of calls at PND2, we can observe that the cross-fostering experiment rescued the alterations in complexity of the calls previously observed in KO male pups (Fig. 5e-f). At both PND8 and PND15 no significant changes were noted between vocalisations from pups fostered by both WT or KO dams (Supplementary Fig. 3). These results suggest that the effects observed with the “normal crossings'' are a combination of GxE effects dependent on the genotype of the pups and of the dam. This data suggests that while WT dams can act as a positive influence on the early development of the pups, the care provided by mutant dams can perturb early neurodevelopment, limit the weight gain in *Gprasp2* mutant pups and perturb USV number and complexity.

## Discussion

Behavioural alterations stemming from genetic disorders may impinge on progeny development, however, little is known on how specific gene mutation affect maternal care. The ability to form and maintain social bonds is a hallmark of social behaviour in mammals and deficits in these processes are a common hallmark in ASD^34,35^. The bonds formed during the perinatal period, particularly the formation of a mother-infant bond, strongly impact social behaviours at later stages of development, making this early postnatal phase a window of vulnerability for future impairments^36,37^, or when taken to the extreme in animals, maternal neglect will result in death.

Poor offspring survival is often paired with either impairments in maternal care, deficits in mammary gland function/ milk production or both^38,39^. In the *Gprasp2* KO dams, we observed a combination of impairments in both maternal nursing behaviours, mammary gland development and *OxtR* gene expression. Since we did not observe dam aggression, and since the surviving pups developed normal body weight despite the significant decrease in litter size, the most parsimonious explanation for the neonatal death would be deficient milk production. In the case of Gprasp2^-/-^ females, they can produce and eject milk, as verified by the presence of milk spots, but more subtle disruptions cannot be excluded. Studies have reported deficits in maternal behaviour in OxtR KO females postpartum, such as longer latencies to retrieve pups^40^. Conditional models of this deletion, where OxtR is only absent in the forebrain, report no striking alterations in maternal behaviours, but a significant increase in pup mortality^38^. Oxytocin is a non-peptide hormone, known for its role in sociosexual behaviours and female reproduction, with an emphasis in parturition and lactation, acting through oxytocin receptors. The expression of these Gq-coupled GPCRs is reported to increase significantly during pregnancy in the brain, particularly in the hypothalamic region^41^, and are thought to support—along with oxytocin, the onset and maintenance of maternal behaviours^42,43^. Altered levels of these receptors have also been reported to lead to an abnormal development of mammary glands in mice, with overall overexpression of OXTR leading to advanced maturation, hyperplasia, ductal distention and early involution of mammary glands in lactation^44,45^**.** The alteration observed in *OxtR* expression levels in primiparous *Gprasp2* KO dams, are in line with the impairments observed in maternal care, mammary gland development and subsequent poor offspring survival.

As the postnatal period is considered a “vulnerability window” susceptible to environmental factors^37^, maternal care and maternal bond become an important focal point to understand the repercussions of challenges during early brain development^46^. Ultrasonic vocalizations emitted by mouse pups are prominent during the first few days of life and decrease throughout development being almost non-existent at weaning^47^. These calls are used by rodents to establish and maintain social contacts with conspecifics, and can convey emotional, behavioural and motivational states of the emitter^48^. Pups USVs also stimulate the innate behaviour from the dams to search for an offspring and retrieve it back to the nest^33,49^. As observed in other animal models of neurodevelopmental disorders^50^, we also uncovered deficits in communication at early development stages in mice deficient in GPRASP2. Nonetheless, the communication impairments observed in KO pups arises from a combination of genetic factors and care provided by mutant dams (heterozygous or knockout). With cross-fostering experiments, we could observe that rearing provided by *Gprasp2* KO dams was not sufficient to lead to significant alterations in the number of the calls emitted by WT pups. In contrast, KO pups reared by WT dams had the vocalizations phenotype rescued.

## Perspectives and significance

The effects of sex-related sociocultural and environmental modifiers, may, to some degree impact the ability of females to be more socially competent at camouflaging autistic symptoms leading to a lack or delay of diagnosis^51,52^. Nonetheless, sex differences in these disorders are often overlooked, leading to a lack of knowledge and a greater misunderstanding of the impact of ASD-linked mutations in social or maternal behaviour in females. Given the sexual dichotomies present in neurodevelopmental disorders, it is important to understand the communalities and differences when exploring genes linked to these same pathologies in the potential for sex-specific alterations in cognition or behaviour.

The hypothalamus has a prominent role in maintaining the body’s homeostasis, being highly involved in the satiety/feeding regulation, hormone regulation, and also having a crucial role in attachment behaviours and parental care^53^. The high expression of GPRASP2 in the hypothalamic region, associated with the increase in body weight and the impairments in maternal care/bonding observed in the *Gprasp2* KO females suggests a significant role for GPRASP2 in hypothalamic function worth further exploration.

As *Gprasp2* is an X-linked gene, to obtain the genotypes required for this study, matings required using *Gprasp2* mutant dams. This raises the question of the importance of the genotype of the dam (and the phenotypical alterations associated with it) when caring for offspring and the impact of those same deficits in the development of the pups. If the animals are cared for by a KO dam, would we see an exacerbation of the adult phenotypes in males and females? Or the opposite, if they were fostered by a WT dam, could it be enough to revert the adult phenotypes? These questions may be worth further exploration to understand the role of early social context in overriding or enhancing the consequence of genetic mutations associated with neurodevelopmental disorders.

## Study limitations

While the changes to OxtR provide an interesting molecular correlate to the observed behavioural changes, this study does not provide a mechanistic explanation for the perturbed expression of these transcripts. Future studies should address if Gprasp2 is directly involved in the regulation of these transcripts, or rather, if disruption of this gene alters circuit function leading to non-cell autonomous changes in the oxytocinergic system. Additionally, we have not performed a deep characterization of the cross-fostered animals later in adult life. As such, while we observe that early developmental differences are rescued by the cross-fostering experiments, it is unclear if, for example, the pronounce memory defects present in Gprasp2 mutant mice would be equally mitigated.

## Conclusions

Our work shows that *Gprasp2* deletion led to alterations in female behaviour, including maternal social interactions. These changes impact neurodevelopment and exacerbate alterations in male KO mice in terms of vocalization and delaying weight gain. More generally, this work shows the importance of better understanding the impact of ASD-link mutations in females, and highlights the relevance of controlling for maternal-driven effects and GxE effects in translational studies.

### Supplementary Information

Supplementary Information.

## Data Availability

All data generated or analysed during this study are included in this published article and its supplementary information files.

## Abbreviations

ASD: Autism spectrum disorder
BCA: Bicinchoninic acid
CER: Cerebellum
CTX: Cortex
DGAV: Direção-Geral da Alimentação e Veterinária
DNA: Deoxyribonucleic Acid
DOC: Sodium deoxycholate
DSM: Diagnostic and statistical manual
ECF: Enhanced fluorescence
EphB2: Ephrin-type B receptor 2
FELASA: Federation of European Laboratory Animal Science Associations
Fmr1: Fragile X messenger Rribonucleoprotein 1
GPCR: G-Protein Couple Receptor
GPRASP2: G-Protein Couple Receptor Associated Sorting Protein 2
HET: Heterozygous
HIPP: Hippocampus
Hprt: Hypoxanthine phosphoribosyltransferase 1
HYP: Hypothalamus
ID: Intellectual disability
KO: Knockout
LED: Light emitting diode
Mecp2: Methyl CpG binding protein 2
Mthfr: Methylenetetrahydrofolate reductase
NaCl: Sodium chloride
NP: Nulliparous
ns: Not significant
ORBEA: Órgãos Responsáveis pelo Bem-Estar dos Animais
Oxt: Oxytocin
OxtR: Oxytocin receptor
PAGE: Polyacrylamide gel electrophoresis
PCR: Polymerase chain reaction
PND: Postnatal day
PP: Primiparous
PVDF: Polyvinylidene fluoride
RIPA: Radioimmunoprecipitation assay
RNA: Ribonucleic acid
RT: Room temperature
SDS: Sodium Dodecyl Sulfate
STR: Striatum
TBS: Tris-buffered saline
Tsc2: Tuberous Sclerosis 2
USV: Ultrasonic Vocalisations
WT: Wild-type

## References

[1] Association AP. Diagnostic and statistical manual of mental disorders 5th ed 2013.

[2] Zeidan J, Fombonne E, Scorah J, Ibrahim A, Durkin MS, Saxena S (2022). Global prevalence of autism: A systematic review update. Autism Res..

[3] Mandy W, Chilvers R, Chowdhury U, Salter G, Seigal A, Skuse D (2012). Sex differences in autism spectrum disorder: Evidence from a large sample of children and adolescents. J. Autism Dev. Disord..

[4] Carter AS, Black DO, Tewani S, Connolly CE, Kadlec MB, Tager-Flusberg H (2007). Sex differences in toddlers with autism spectrum disorders. J. Autism Dev. Disord..

[5] Supekar K, Iyer T, Menon V (2017). The influence of sex and age on prevalence rates of comorbid conditions in autism. Autism Res..

[6] Rodgaard EM, Jensen K, Miskowiak KW, Mottron L (2021). Autism comorbidities show elevated female-to-male odds ratios and are associated with the age of first autism diagnosis. Acta Psychiatr. Scand..

[7] Loomes R, Hull L, Mandy WPL (2017). What is the male-to-female ratio in autism spectrum disorder? A systematic review and meta-analysis. J Am. Acad. Child Adolesc. Psychiatr..

[8] Skuse DH (2000). Imprinting, the X-chromosome, and the male brain: explaining sex differences in the liability to autism. Pediatr Res..

[9] Baron-Cohen S, Knickmeyer RC, Belmonte MK (2005). Sex differences in the brain: Implications for explaining autism. Science.

[10] Zhang Y, Li N, Li C, Zhang Z, Teng H, Wang Y (2020). Genetic evidence of gender difference in autism spectrum disorder supports the female-protective effect. Transl. Psychiatr..

[11] Assali A, Cho JY, Tsvetkov E, Gupta AR, Cowan CW (2021). Sex-dependent role for EPHB2 in brain development and autism-associated behavior. Neuropsychopharmacology..

[12] Ferreira H, Sousa AC, Sereno J, Martins J, Castelo-Branco M, Goncalves J (2022). Sex-dependent social and repetitive behavior and neurochemical profile in mouse model of autism spectrum disorder. Metabolites.

[13] Giovanniello J, Ahrens S, Yu K, Bo L (2021). Sex-specific stress-related behavioral phenotypes and central amygdala dysfunction in a mouse model of 16p112 microdeletion. Biol. Psychiatr. Glob. Open Sci..

[14] Santos S, Martins B, Sereno J, Martins J, Castelo-Branco M, Goncalves J (2023). Neurobehavioral sex-related differences in Nf1(+/-) mice: female show a "camouflaging"-type behavior. Biol. Sex Differ..

[15] Levav-Rabkin T, Blumkin E, Galron D, Golan HM (2011). Sex-dependent behavioral effects of Mthfr deficiency and neonatal GABA potentiation in mice. Behav. Brain Res..

[16] Ding Q, Sethna F, Wang H (2014). Behavioral analysis of male and female Fmr1 knockout mice on C57BL/6 background. Behav. Brain Res..

[17] Grabrucker S, Pagano J, Schweizer J, Urrutia-Ruiz C, Schon M, Thome K (2021). Activation of the medial preoptic area (MPOA) ameliorates loss of maternal behavior in a Shank2 mouse model for autism. EMBO J..

[18] Krishnan K, Lau BY, Ewall G, Huang ZJ, Shea SD (2017). MECP2 regulates cortical plasticity underlying a learned behaviour in adult female mice. Nat. Commun..

[19] Young DM, Schenk AK, Yang SB, Jan YN, Jan LY (2010). Altered ultrasonic vocalizations in a tuberous sclerosis mouse model of autism. Proc. Natl. Acad. Sci. USA.

[20] Basu SN, Kollu R, Banerjee-Basu S (2009). AutDB: A gene reference resource for autism research. Nucleic Acids Res..

[21] Gong L, Yan Y, Xie J, Liu H, Sun X (2012). Prediction of autism susceptibility genes based on association rules. J. Neurosci. Res..

[22] Butler MG, Rafi SK, Hossain W, Stephan DA, Manzardo AM (2015). Whole exome sequencing in females with autism implicates novel and candidate genes. Int. J. Mol. Sci..

[23] Zhou J, Goldberg EM, Leu NA, Zhou L, Coulter DA, Wang PJ (2014). Respiratory failure, cleft palate and epilepsy in the mouse model of human Xq22.1 deletion syndrome. Hum. Mol. Genet..

[24] Heydorn A, Sondergaard BP, Ersboll B, Holst B, Nielsen FC, Haft CR (2004). A library of 7TM receptor C-terminal tails. Interactions with the proposed post-endocytic sorting proteins ERM-binding phosphoprotein 50 (EBP50), N-ethylmaleimide-sensitive factor (NSF), sorting nexin 1 (SNX1), and G protein-coupled receptor-associated sorting protein (GASP). J. Biol. Chem..

[25] Edfawy M, Guedes JR, Pereira MI, Laranjo M, Carvalho MJ, Gao X (2019). Abnormal mGluR-mediated synaptic plasticity and autism-like behaviours in Gprasp2 mutant mice. Nat. Commun..

[26] Peca J, Feliciano C, Ting JT, Wang W, Wells MF, Venkatraman TN (2011). Shank3 mutant mice display autistic-like behaviours and striatal dysfunction. Nature.

[27] Deacon RM (2006). Assessing nest building in mice. Nat. Protoc..

[28] Scattoni ML, Gandhy SU, Ricceri L, Crawley JN (2008). Unusual repertoire of vocalizations in the BTBR T+tf/J mouse model of autism. PLoS One.

[29] Bosch OJ (2013). Maternal aggression in rodents: Brain oxytocin and vasopressin mediate pup defence. Philos. Trans. R Soc. Lond. B Biol. Sci..

[30] Ricceri L, Moles A, Crawley J (2007). Behavioral phenotyping of mouse models of neurodevelopmental disorders: Relevant social behavior patterns across the life span. Behav. Brain Res..

[31] D'Amato FR, Scalera E, Sarli C, Moles A (2005). Pups call, mothers rush: does maternal responsiveness affect the amount of ultrasonic vocalizations in mouse pups?. Behav. Genet..

[32] Cohen-Salmon C, Carlier M, Roubertoux P, Jouhaneau J, Semal C, Paillette M (1985). Differences in patterns of pup care in mice. V-Pup ultrasonic emissions and pup care behavior. Physiol. Behav..

[33] Noirot E (1972). The onset of maternal behavior in rats, hamsters, and mice a selective review. Adv. Study Behav. Acad. Press.

[34] Tunçgenç, B., Carolyn Koch, Inge-Marie Eigsti, Stewart H. Mostofsky. Mimicry and social affiliation with virtual partner are decreased in autism. Res. Autism Spectr. Disord. **100** (2023). 10.1016/j.rasd.2022.102073 PMC12965197 41798060

[35] McKenzie R, Dallos R (2017). Autism and attachment difficulties: Overlap of symptoms, implications and innovative solutions. Clin Child Psychol Psychiatry..

[36] Rilling JK, Young LJ (2014). The biology of mammalian parenting and its effect on offspring social development. Science.

[37] Andersen SL (2003). Trajectories of brain development: Point of vulnerability or window of opportunity?. Neurosci. Biobehav. Rev..

[38] Macbeth AH, Stepp JE, Lee HJ, Young WS, Caldwell HK (2010). Normal maternal behavior, but increased pup mortality, in conditional oxytocin receptor knockout females. Behav. Neurosci..

[39] Bartsch VB, Lord JS, Diering GH, Zylka MJ (2020). Mania- and anxiety-like behavior and impaired maternal care in female diacylglycerol kinase eta and iota double knockout mice. Genes Brain Behav..

[40] Takayanagi Y, Yoshida M, Bielsky IF, Ross HE, Kawamata M, Onaka T (2005). Pervasive social deficits, but normal parturition, in oxytocin receptor-deficient mice. Proc. Natl. Acad. Sci. USA.

[41] Mitre M, Marlin BJ, Schiavo JK, Morina E, Norden SE, Hackett TA (2016). A distributed network for social cognition enriched for oxytocin receptors. J. Neurosci..

[42] Feldman R (2017). The neurobiology of human attachments. Trends Cogn. Sci..

[43] Insel TR, Shapiro LE (1992). Oxytocin receptor distribution reflects social organization in monogamous and polygamous voles. Proc. Natl. Acad. Sci. USA.

[44] Li D, San M, Zhang J, Yang A, Xie W, Chen Y (2021). Oxytocin receptor induces mammary tumorigenesis through prolactin/p-STAT5 pathway. Cell Death Dis..

[45] Li, D., Ji, Y., Zhao, C., Yao, Y., Yang, A., Jin, H., et al. OXTR overexpression leads to abnormal mammary gland development in mice. J. Endocrinol. (2018). 10.1530/JOE-18-0356 30089682

[46] Curley JP, Champagne FA (2016). Influence of maternal care on the developing brain: Mechanisms, temporal dynamics and sensitive periods. Front. Neuroendocrinol..

[47] Scattoni ML, Crawley J, Ricceri L (2009). Ultrasonic vocalizations: a tool for behavioural phenotyping of mouse models of neurodevelopmental disorders. Neurosci. Biobehav. Rev..

[48] Wohr M, Schwarting RK (2013). Affective communication in rodents: ultrasonic vocalizations as a tool for research on emotion and motivation. Cell Tissue Res..

[49] Ehret G, Koch M, Haack B, Markl H (1987). Sex and parental experience determine the onset of an instinctive behavior in mice. Naturwissenschaften.

[50] Moy SS, Nadler JJ (2008). Advances in behavioral genetics: Mouse models of autism. Mol. Psychiatr..

[51] Kreiser NL, White SW (2014). ASD in females: are we overstating the gender difference in diagnosis?. Clin. Child Fam. Psychol. Rev..

[52] Corbett BA, Schwartzman JM, Libsack EJ, Muscatello RA, Lerner MD, Simmons GL (2021). Camouflaging in autism: Examining sex-based and compensatory models in social cognition and communication. Autism Res..

[53] Saper CB, Lowell BB (2014). The hypothalamus. Curr Biol..

