# Supplementary material for: Maternal behaviours disrupted by Gprasp2 deletion modulate neurodevelopmental trajectory in progeny
Source: Sci Rep. 2024 May 31;14:12484. doi: 10.1038/s41598-024-62088-x (PMC11139669; doi:10.1038/s41598-024-62088-x)
Supplement: Supplementary file 1 — Supplementary Information. [file 41598_2024_62088_MOESM1_ESM.pdf]

Supplementary Material

Maternal behaviours disrupted by Gprasp2 deletion modulate neurodevelopmental trajectory in progeny

Marta I. Pereira; Mariana Laranjo; Marcos Gomes; Mohamed Edfawy; João Peça

Supplementary Figures & Tables

Supplementary Table 1. Phenotypical comparison between male and female *Gprasp2* KO mice when comparing with same-sex wild-type control.

Parameter	*Gprasp2* $^{-/y}$ males (from Ref. 25)	*Gprasp2* $^{-/-}$ females
Body weight	• Significant increase in body weight starting at 12 weeks-old	• Significant increase in body weight starting at 16 weeks-old
Anxiety-Like Behaviours	• Decrease in anxiety-like behaviours (open field test and elevated plus maze)	• No differences noted (open field test and elevated plus maze)
Locomotor activity	• No differences noted (distance travelled open field)	• No differences noted (distance travelled open field)
Working and spatial memory	• Impairment in working spatial memory (T-maze for spontaneous alternation, Barnes Maze, Novel object recognition)	• Impairment in working spatial memory (T-maze for spontaneous alternation)
Repetitive behaviours	• Decrease in number of marbles buried<br>• Impaired nest building	• Decrease in number of marbles buried<br>• No gross differences in nest building
Social Interaction	• Decreased preference for a social partner<br>• Decreased preference for a novel conspecific<br>• Increase in non-reciprocal interactions with a non-familiar conspecific	• No gross differences in preference for a social partner<br>• Decreased preference for a novel conspecific<br>• Tendency for increase in interactions with non-familiar conspecific
Aggression/ Social dominance	• Increase in social dominance (tube test)	• No significant alterations in maternal aggression

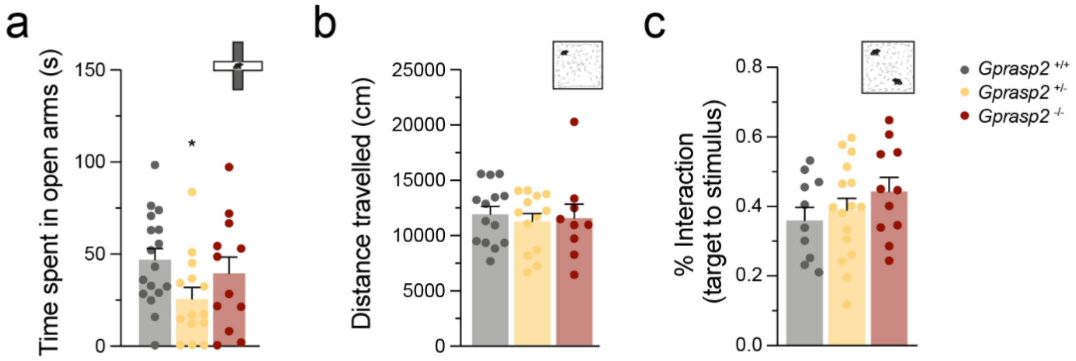

Supplementary Figure 1. *Gprasp2*$^{-/-}$ and *Gprasp2*$^{+/-}$ females show no significant impairments in anxiety and locomotor activity while presenting a tendency for altered social interactions. **a.** No alterations are observed in *Gprasp2*$^{-/-}$ and *Gprasp2*$^{+/-}$ females in the elevated plus maze; WT n=17, HET n=14, KO n=12. **b.** Females show no impairments in locomotor activity in the open field test; WT n=14, HET n=13, KO n=9. **c.** In the social dyadic test, *Gprasp2*$^{-/-}$ females show an increased tendency to explore a social partner when compared to WT controls WT n=6, HET n=11, KO n=8. All data are presented as means ± s.e.m.

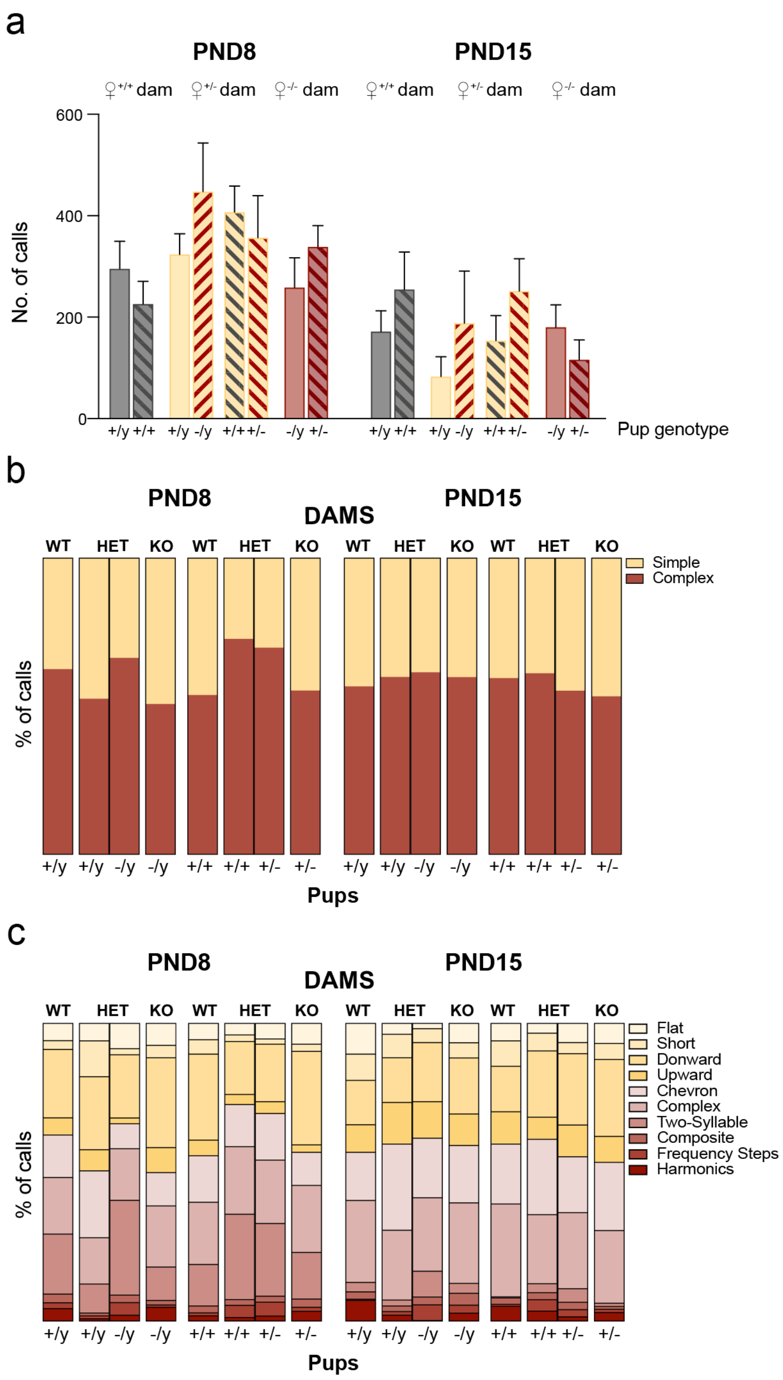

Supplementary Figure 2. Alterations observed at PND2 are no longer seen at PND8 and PND15**. a.** Pups cared for by HET dams show an overall increase in the number of calls at PND8, which is no longer present at PND15. However, no significant differences can be noted between genotype-matched pups. **b-c.** As for the complexity of these calls, a sex-specific decrease is observed in complex calls in males, specifically in *Gprasp2*$^{+/y}$ pups from HET dams and *Gprasp2*$^{-/y}$ pups from KO dams at PND8 (increase in downward calls (simple) when compared to WT pups). At PND15, no alterations in the complexity of calls can be noted. (+/y)$^{\text{WT dams}}$ n=15; (+/+)$^{\text{WT dams}}$ n=12; (+/y)$^{\textit{Gprasp2}\text{ +/- dams}}$ n=13; (-/y)$^{\textit{Gprasp2}\text{ +/- dams}}$ n=5; (+/+)$^{\textit{Gprasp2}\text{ +/- dams}}$ n=14; (+/-)$^{\textit{Gprasp2}\text{ +/- dams}}$ n=9; (-/y)$^{\textit{Gprasp2}\text{ -/- dams}}$ n=12; (+/-)$^{\textit{Gprasp2}\text{ -/- dams}}$ n=7. Data presented in (a) are displayed as means ± s.e.m.

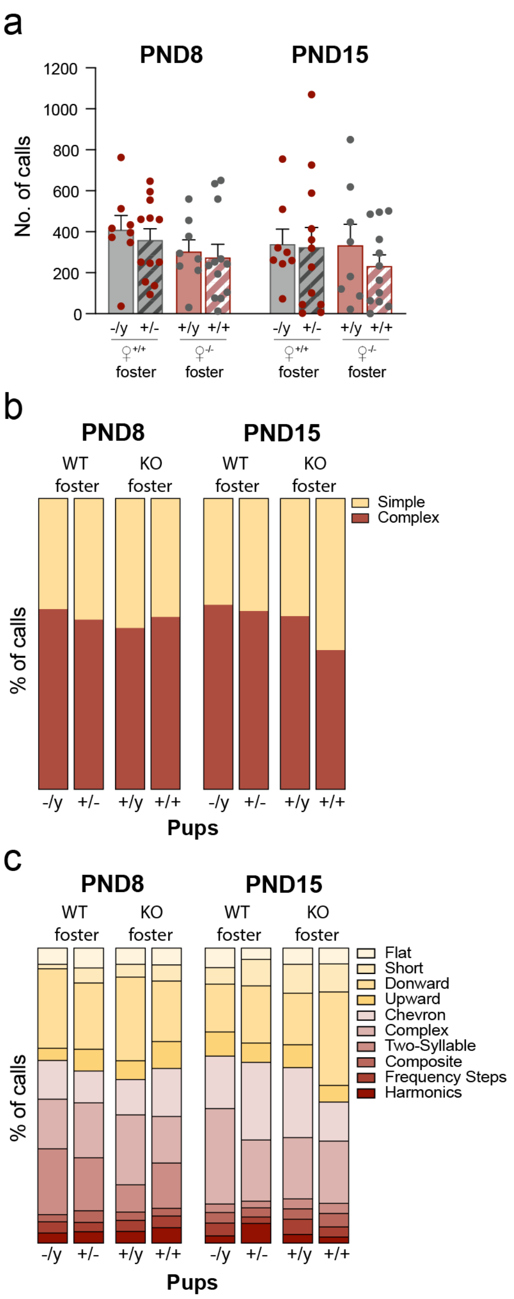

Supplementary Figure 3. Cross fostering leads to a normalisation of the number and complexity of calls in pups cared for either WT or KO fosters dams. **a-c.** After cross fostering experiment, no changes can be noted between the different pups both in terms of number (**a)** and complexity of the calls (**b-c**) independently of sex and foster genotype. (+/y;) n=8; (+/+) n=15; (-/y) n=8; (+/-) n=15. Data presented in (a) are displayed as means ± s.e.m.

Western blot Figure 1A

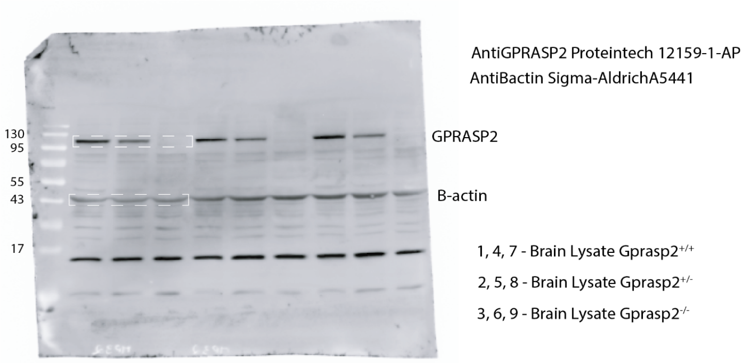

Western blot Figure 1B

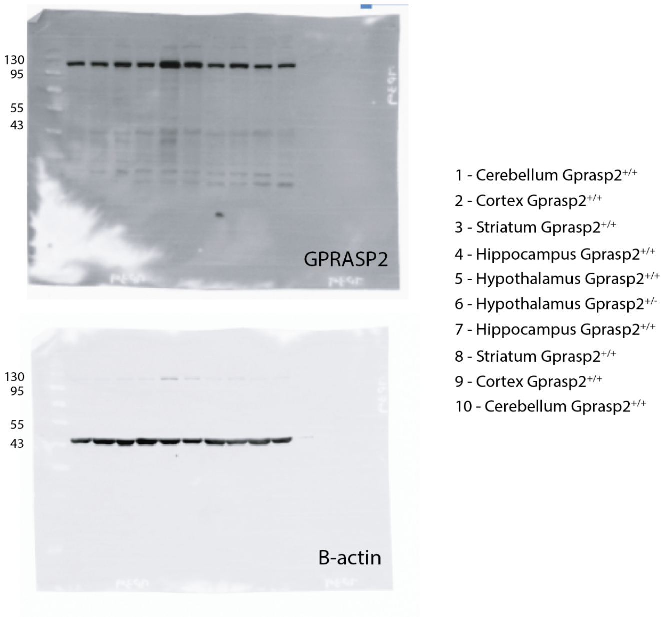

Supplementary Figure 4. Western blot images for the data reported in Figure 1a and 1b, respectively, along with the identification of samples and antibodies used.
